# Functional Analysis of *PsHMGR1* and *PsTPS1* Related to Floral Terpenoids Biosynthesis in Tree Peony

**DOI:** 10.3390/ijms252212247

**Published:** 2024-11-14

**Authors:** Bo Ma, Zi-Yao Li, Rong-Chen Li, Mei-Chen Xu, Zhen-Quan Wang, Ping-Sheng Leng, Zeng-Hui Hu, Jing Wu

**Affiliations:** 1 College of Landscape Architecture, Beijing University of Agriculture, Beijing 102206, China; mabohy@163.com (B.M.); liziiyao@163.com (Z.-Y.L.); 15613278310@163.com (R.-C.L.); 13240090816@163.com (M.-C.X.); wang17667846990@163.com (Z.-Q.W.); lengpsh@tom.com (P.-S.L.); 2 Beijing Laboratory of Urban and Rural Ecological Environment, Beijing 102206, China; 3 Ancient Tree Health and Culture Engineering Technology Research Center, National Forestry and Grassland Administration, Beijing 102206, China

**Keywords:** *Paeonia suffruticosa* ‘Oukan’, floral fragrance, terpenoids, TPS, HMGR

## Abstract

Tree peony (*Paeonia suffruticosa*), as a popular ornamental plant worldwide, has a unique floral fragrance, and it is important in the pollination, ornamental, food, and fragrance product industries. However, the underlying molecular mechanisms for the synthesis of floral fragrance terpenoids in tree peony are not well understood, constraining their exploitation. *P. suffruticosa* ‘Oukan’ produces strong floral fragrance terpenoids with high ornamental value and excellent stress resistance and is considered a valuable model for studying tree peony floral fragrance formation. Based on transcriptome data analysis, the *PsHMGR1* and *PsTPS1* genes associated with floral terpene synthesis were cloned. Then, PsHMGR1 and PsTPS1 were functionally characterized by amino acid sequence analysis, multiple sequence alignment, phylogenetic tree construction, qRT-PCR, and transgenic assay. PsHMGR1 contains two transmembrane structures and a conserved HMG-CoA_reductase_class I domain, and PsTPS1 belongs to TPS-a subfamily. The qRT-PCR analysis showed that the expression levels of *PsHMGR1* and *PsTPS1* increased and then decreased at different flower development stages, and both were significantly higher in flowers than in roots, stems, and leaves. In addition, the linalool content in *PsHMGR1* transgenic lines was significantly higher than that of WT. Germacrene D, which was not found in WT, was detected in the flowers of PsTPS1 transgenic lines. These results indicate that PsHMGR1 and PsTPS1 promote terpene synthesis in plants and provide ideas for the molecular mechanism of enhancing terpene synthesis in tree peony floral fragrance.

## 1. Introduction

Releasing floral fragrance is an important feature of flowering plants. Floral fragrance, a secondary metabolite released from flowers, is a typical component of plant volatile organic compounds (VOCs), which is crucial in physiological and ecological processes [1,2]. For the plants, floral fragrance not only helps them to reproduce and improve resistance but also acts as communication signal [3]. Meanwhile, as an important indicator of flower quality, the improvement of floral fragrance traits is significant for enhancing the flowers ornamental and economic value [4]. Compared to flower color and shape, floral fragrance is more likely to influence consumer choice; however, the development of scent in many flower varieties has often been neglected in traditional breeding programs [5,6,7,8]. In addition, floral volatile compounds have been used in a wide range of applications, such as food, pharmaceuticals, additives, and flavoring products [3,9]. Based on the biosynthetic origin in plants, floral volatile compounds are generally classified into three groups: terpenoids, phenylpropanoids/benzenoids, and fatty acid derivatives [10,11]. Terpenoids, as the largest group of them, are one of the main components that make up the floral fragrance of ornamental plants [12,13]. Studies have confirmed the predominance of terpenoids in the floral fragrance in strongly scented *Lilium* species [10,14], *Chrysanthemum morifolium* [15], *Aster* species [16], *Phalaenopsis* species [17], *Fressia hybrida* [18], *Rosa damescena* [7], and *Osmanthus fragrans* [19,20].

Terpenoids in plants have been reported to be synthesized through two compartmentally separated metabolic pathways: the mevalonate (MVA) pathway in the cytosol and the methylerythritol phosphate (MEP) pathway in plastids [8,21]. And these two pathways are not completely independent, and there is cross-talk between them [11,22]. 3-hydroxy-3-methyl-glutaryl CoA (HMG-CoA) reductase (HMGR), the first key rate-limiting enzyme in the MVA pathway, plays a very important role in controlling the flow of carbon sources in the metabolic pathway [23]. Moreover, catalyzing the synthesis of HMG-CoA by HMGR is a critical regulatory step in the biosynthesis of terpenoids [24,25,26,27]. In recent years, more and more *HMGR* genes from different plants have been isolated and cloned, and their expression patterns have been analyzed and found to be highly expressed in metabolite-enriched organs [23,25]. The *HbHMGR1* gene is expressed at significantly higher levels in latex than in leaves, phloem, and xylem and may play an important role in natural rubber biosynthesis in *Hevea brasiliensis* [28]. Both *PgHMGR1* and *PgHMGR2* genes are expressed at higher levels in 3- and 6-year-old ginseng roots than in other organs, suggesting that *PgHMGRs* are involved in the biosynthesis of ginsenosides [29]. The functions of *HMGRs* in terpene biosynthesis have been investigated in model plants and medicinal plants [26,27,30,31,32]. In *Artemisia annua*, the artemisinin content of *hmgr* transgenic lines is higher than in non-transgenic lines [33]. Transient expression of the *AmHMGR* gene from *Antirrhinum majus* in tomato results in a 5.7-fold and 1.8-fold increase in monoterpene nerolidol and linalool, respectively [34]. However, the characterization of the *HMGR* gene of ornamental plants needs to be further explored.

Terpene synthase (TPS), a key enzyme downstream of the terpene biosynthesis pathway, is directly involved in the synthesis of terpene products. TPS largely determines the structural and functional diversity of terpenoids and plays a crucial role in the overall terpene synthesis process [4,35,36,37]. Therefore, the functional characterization of *TPS* in plants has received increasing attention. In recent years, *TPS* genes have been gradually isolated from *Arabidopsis thaliana* [37], *Solanum lycopersicum* [38], *Freesia* × *hybrida* [8,18], *Lilium* ‘Siberia’ [39], *Lathyrus odoratus* [35], *Cymbidium faberi* [40], and *Chrysanthemum morifolium* [41] and demonstrated their roles in terpene volatile biosynthesis. Based on the functions and homology, plant TPSs have been clustered into seven subfamilies (TPS-a to TPS-h) [18]. Among them, members of the TPS-a/b/g subfamilies are responsible for the majority of floral volatile monoterpene and sesquiterpene compounds, whose emissions are found to be significantly correlated with the expression of *TPSs* [8,18,35,41]. For example, the temporal expression pattern of the *FhTPS1* (TPS-b) gene during flower development in *Freesia* petals is consistent with linalool emission [18]. The *LoTPS4* (TPS-a) gene expressed at a high level in petal at the S4 period has been demonstrated to catalyze the production of ocimene, myrcene, and farnesene in *L. odoratus* [35]. Therefore, it is of great significance to study the expression pattern and synthesis mechanism of *TPS* genes to reveal the biosynthesis of floral terpene volatiles.

*Paeonia suffruticosa*, a woody flower with high ornamental and applied value, is one of the traditional Chinese famous flowers and enjoys the reputation of ‘national beauty and heavenly fragrance’ [42,43,44]. Tree peony is famous for its large and colorful flowers, rich flower shapes, and strong fragrance [45,46]. Its floral fragrance, as an important ornamental trait and evaluation index of flower food flavor, greatly influences the consumer market [47]. In addition, floral scent is often neglected in breeding, resulting in a considerable number of tree peony cultivars with an overly strong aroma and a few varieties suitable for flower forcing with an unpleasant scent [43]. However, compared with research on flower shape and color, studies on floral scent, especially the molecular synthesis mechanism, are still lagging behind, which limits the tree peony commercial development [48,49,50]. Therefore, it is important to understand the types of substances and the functions of key synthetic genes for tree peony floral scent and to breed new tree peony varieties with pleasant aromas using modern breeding techniques [45]. Currently, studies related to the tree peony floral fragrance mainly focus on the volatile components [42,51], among which terpenoids account for a relatively high proportion [44,46,48,52], such as ocimene, linalool, citronellol, and germacrene D [44,52,53]. So far, though several terpene synthesis-related enzyme genes have been cloned, including *PdDXS* in *P. delavayi* [54], *PsDXS* [55], *PsDXR*, and *PsMCS* in cultivars ‘Oukan’ [56], *PsGPPS* [46], *PsGDS* [53], *PsTPS14* [57], *PsuLIS* [45], and five *PsTPSs* (*PsTPS1*~*PsTPS5*) [52] in ‘High Noon’, fewer genes are functionally characterized. Transient overexpression of *PsTPS14* in tobacco leaves and the lightly scented tree peony flower ‘Fengdan’ increases enzyme activity and releases amounts of linalool [57]. And *PsTPS1* and *PsTPS4* are shown to involve in linalool synthesis after in vitro enzyme activity analysis and transient overexpression in tobacco leaves [52]. The isolation and functional characterization of terpene synthesis pathway genes are very important in the study of tree peony floral fragrance biosynthesis mechanism; however, the relevant research foundation is relatively weak. Therefore, it is particularly important to clone these genes and perform functional analyses in tree peony.

The strongly scented tree peony cultivar ‘Oukan’ has outstanding ornamental value and is an excellent material for the study of floral fragrance biosynthesis. However, little is known about the isolation and functional exploration of terpene biosynthetic pathway genes in ‘Oukan’. Our previous study has demonstrated that terpenoids are the main components in the floral fragrance of ‘Oukan’, and *PsHMGR1* and *PsTPS* may play an important role in terpenoid synthesis [42]. Therefore, in this study, we cloned *PsHMGR1* and *PsTPS1* genes, analyzed temporal expression patterns, and transgenically characterized their functions in tobacco. This result provides important genetic resources and biological insights into the mechanism of volatile terpene biosynthesis in tree peony flowers and floral scent breeding.

## 2. Results

### 2.1. Cloning and Bioinformatics Analysis of PsHMGR1

**Figure 1:**
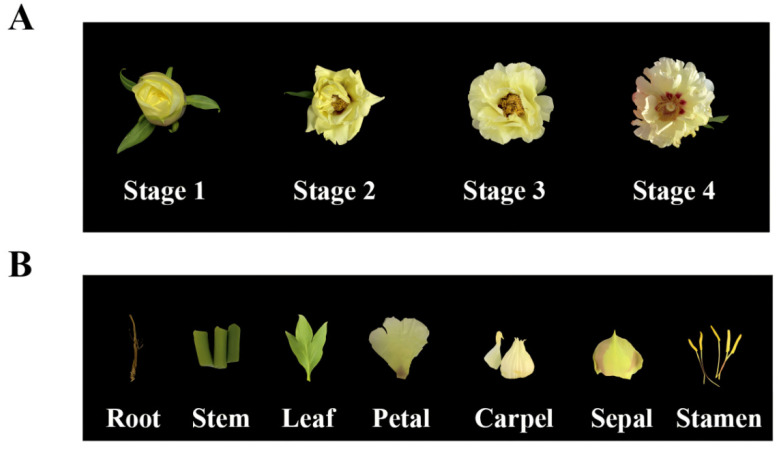
Photos of cultivar *P. suffruticosa* ‘Oukan’. (**A**) Four different flower developmental stages. Stage 1, bud brusting stage; Stage 2, initial flowering stage; Stage 3, full blooming stage; Stage 4, flower withering stage. (**B**) Different organs and tissues in Stage 3.

**Figure 2:**
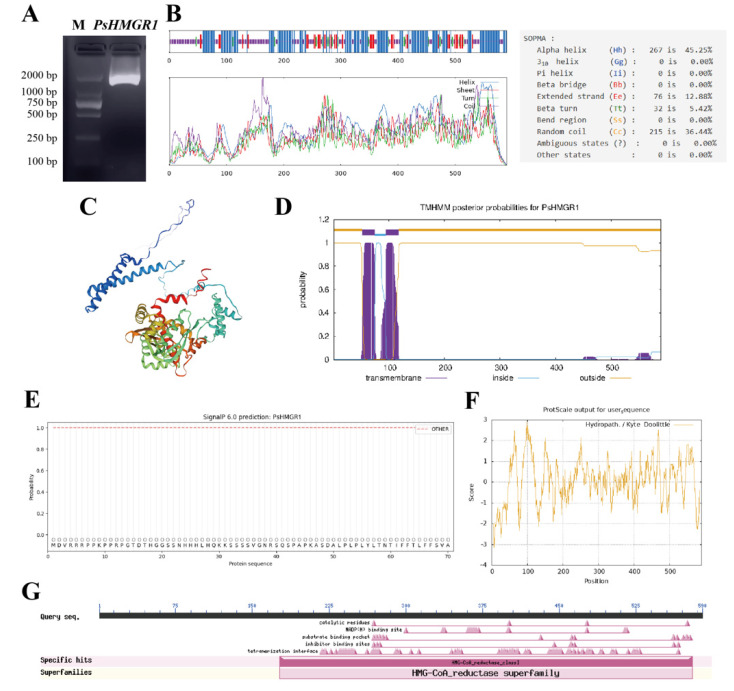
The electrophoresis map of the *PsHMGR1* gene and PsHMGR1 protein characterization. (**A**) Electrophoresis map of *PsHMGR1* gene. M, marker. (**B**) Secondary structure prediction, (**C**) 3D model building, (**D**) transmembrane helices prediction, (**E**) hydrophobicity or hydrophilicity analysis, (**F**) signal peptide analysis, and (**G**) conserved domains analysis of PsHMGR1 protein.

Based on the obtained *P. suffruticosa* ‘Oukan’ (Figure 1) transcriptome data, the cloned *PsHMGR1* had an open reading frame (ORF) of 1773 bp (Figure 2A), encoding 590 amino acids (Figure S1). The secondary structure results showed that PsHMGR1 mostly included 267 α-helices (45.25%), followed by 215 random coils (36.44%), 76 extended strands (12.88%), and 32 β-turns (5.42%) (Figure 2B). The theoretical isoelectric point (pI) of PsHMGR1 was 6.9, the theoretical relative molecular weight was 63.34 kD, the total number of negatively charged residues (Asp + Glu) was 58, and the total number of positively charged residues (Arg + Lys) was 57. The tertiary structure modeling of PsHMGR1 protein was modeled on the HMGR of *Populus trichocarpa* with 80.68% similarity and a GMQE value of 0.83 (Figure 2C). The transmembrane structure analysis suggested the PsHMGR1 protein contains two transmembrane structural domains located at amino acids 52–74 and 95–117, respectively (Figure 2D). Moreover, there were no protein signal peptides analyzed in PsHMGR1 (Figure 2E). And PsHMGR1 was found to be an unstable hydrophobic protein with a grand average of hydropathicity (GRAVY) of 0.061, an aliphatic index of 95.53, and an instability index of 49.67 (Figure 2F). The conserved domain analysis of PsHMGR1 indicated that one core specific sequence was HMG-CoA_reductase_class I, which belonged to the HMG-CoA_reductase superfamily and catalyzed the synthesis of coenzyme A and mevalonate in the isoprenoid synthesis multifunctional domain (Figure 2G).

### 2.2. Phylogenetic Tree and Multiple Sequence Alignment Analysis of PsHMGR1

**Figure 3:**
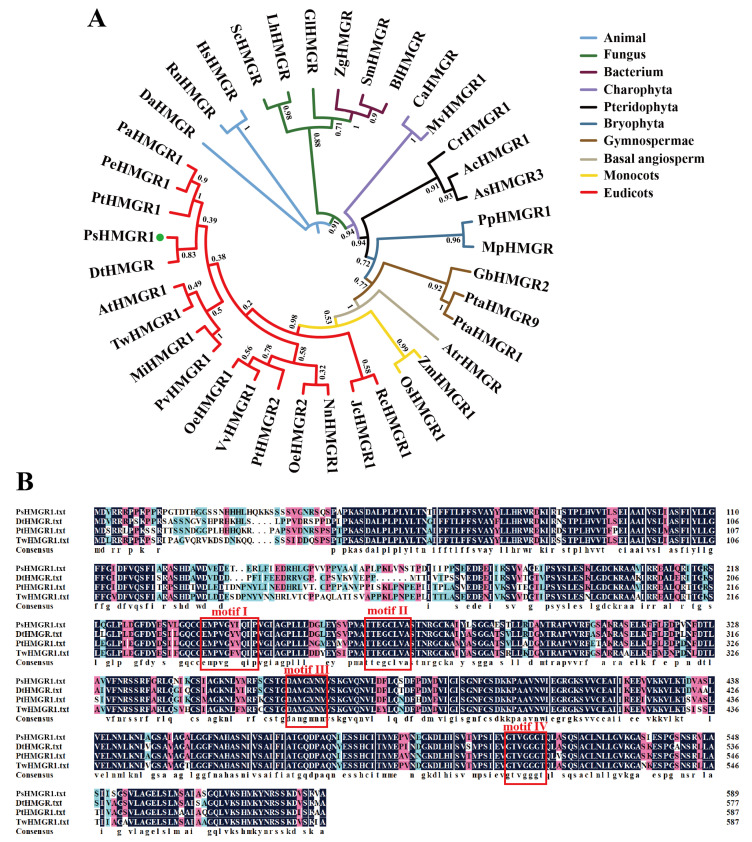
Phylogenetic tree and multiple sequence alignment of HMGR proteins. (**A**) Phylogenetic tree of PsHMGR1 and HMGR protein from other 37 plant species, using the IQ-TREE of TBtools V2.136 software. Bootstrap values are shown as a percentage of 5000 replicates. PsHMGR1 is marked with a green dot. Clades of various species are highlighted with different color lines. Dt, *Dillenia turbinata*; Tw, *Tripterygium wilfordii*; Rc, *Ricinus communis*; Pt, *Populus trichocarpa*; Nn, *Nelumbo nucifera*; Pe, *Populus euphratica*; Mi, *Mangifera indica*; Pa, *Populus alba*; Pv, *Pistacia vera*; Jc, *Jatropha curcas*; Ca, *Chlorokybus atmophyticus*; Mv, *Mesostigma viride*; Pp, *Physcomitrella patens*; Mp, *Marchantia polymorpha*; As, *Alsophila spinulosa*; Cr, *Ceratopteris richardii*; Ac, *Adiantum capillus*; Pta, *Pinus tabuliformis*; Gb, *Ginkgo biloba*; Atr, *Amborella trichopoda*; Os, *Oryza sativa*; Zm, *Zea mays*; Oe, *Olea europaea*; Vv, *Vitis vinifera*; At, *Arabidopsis thaliana*; Hs, *Homo sapiens*; Rn, *Rattus norvegicus*; Da, *Drosophila albomicans*; Sc, *Saccharomyces cerevisiae*; Gl, *Ganoderma lucidum*; Lh, *Lachnellula hyalina*; Zg, *Zobellia galactanivorans*; Sm, *Streptomyces malaysiensis*; Bl, *Brevibacterium linens*. (**B**) Alignment and analysis of PsHMGR1 with HMGR protein sequences of *D. turbinata*, *P. trichocarpa*, and *T. wilfordii*. Motif I, E(M/L)P(V/I)GY(V/I)Q(I/L)P; motif II, TTEGCLVA; motif III, DAMGMNM; motif IV, GTVGGGT. Accession information of HMGR proteins of other 37 plant species is detailed in Supplemental Table S1.

The phylogenetic tree was constructed using all 38 HMGR protein sequences from 36 species, including animals, fungi, bacteria, and plants, to explore the evolution of PsHMGR1 (Figure 3A). The phylogenetic tree results were in agreement with the traditional taxonomy, in which PsHMGR1 clustered in the eudicots lineage (Figure 3A). And PsHMGR1 had shorter branch length within the eudicots group, suggesting that PsHMGR1 was phylogenetically distinct and later than other species (Figure 3A). Compared to other homologous proteins, PsHMGR1 was more closely related to the evolution of the DtHMGR protein (HMGR of *Dillenia turbinata*) and also clustered on the same branch as PtHMGR1 (HMGR of *P. trichocarpa*), PeHMGR1 (HMGR of *P. euphratica*), and PaHMGR1 (HMGR of *P. alba*) (Figure 3A).

Multiple sequence alignment of amino acids revealed that PsHMGR1 contained two HMG-CoA binding motifs (motif I, EMPVGYVQIP; motif II, TTEGCLVA) and two NADP(H) binding motifs (motif III, DAMGMNM; motif IV, GTVGGGT) (Figure 3B). And PsHMGR1 shared the most amino acid sequence identity (83.39%) with DtHMGR, and 80.71% and 80.41% with PtHMGR1 and TwHMGR1, respectively (Figure 3B). These results indicated that PsHMGR1 was highly conserved in the evolutionary process.

### 2.3. Cloning and Bioinformatics Analysis of PsTPS1

**Figure 4:**
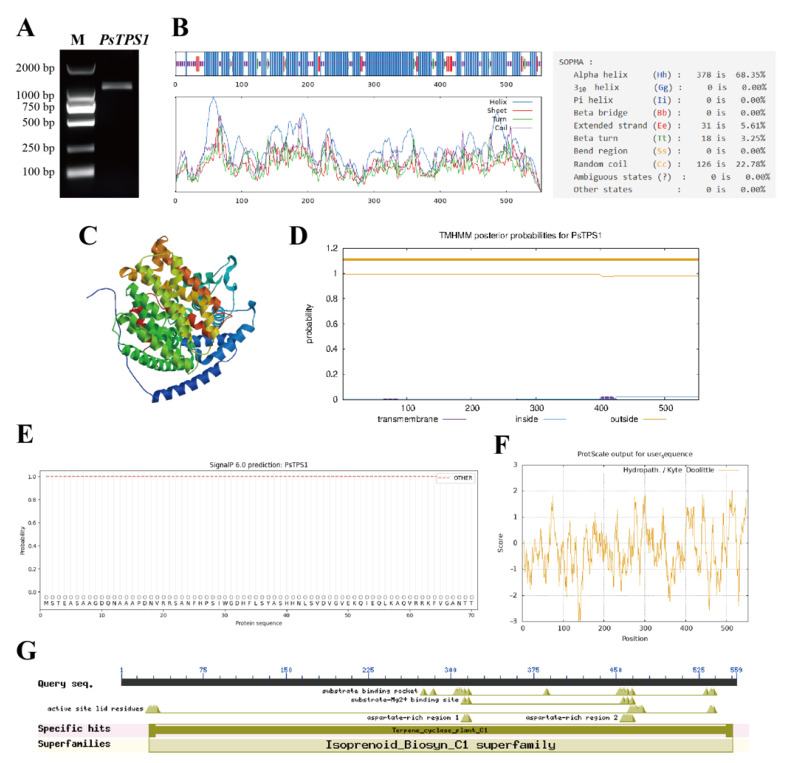
The electrophoresis map of the *PsTPS1* gene and PsTPS1 protein characterization. (**A**) Electrophoresis map of *PsTPS1* gene. M, marker. (**B**) Secondary structure prediction, (**C**) 3D model building, (**D**) transmembrane helices prediction, (**E**) hydrophobicity or hydrophilicity analysis, (**F**) signal peptide analysis, and (**G**) conserved domains analysis of PsTPS1 protein.

The complete ORF of *PsTPS1* was 1662 bp (Figure 4A) and encoded 553 amino acids (Figure S2). The theoretical relative molecular weight of the PsTPS1 protein was 63.63 kD, and the theoretical pI was 5.25. The total number of negatively charged residues (Asp + Glu) was 73, and the total number of positively charged residues (Arg + Lys) was 53. The secondary structure predicted that PsTPS1 contained 378 α-helix (68.35%), 126 random coil (22.78%), 31 extended strands (5.61%), and 18 β-turns (3.25%) (Figure 4B). The GMEQ value for tertiary structure modeling was 0.92, and the results suggested that PsTPS1 protein had a high similarity of 72.54% with the TPS01 model of *Liquidambar formosana* (Figure 4C). TMHMM and signal peptide analyses also showed PsTPS1 had no signal peptide and transmembrane structural (Figure 4D,E). And PsTPS1 protein exhibited a hydrophilic and unstable nature with a GRAVY value of −0.237, aliphatic index of 92.06, and instability index of 46.39 (Figure 4F). The conserved domains analysis revealed that PsTPS1 contained a Terpene_cyclase_plant_C1 (Isopernoid_Biosyn_C1 surperfamily) domain with terpene synthase activity (Figure 4G).

### 2.4. Phylogenetic Tree and Multiple Sequence Alignment Analysis of PsTPS1

**Figure 5:**
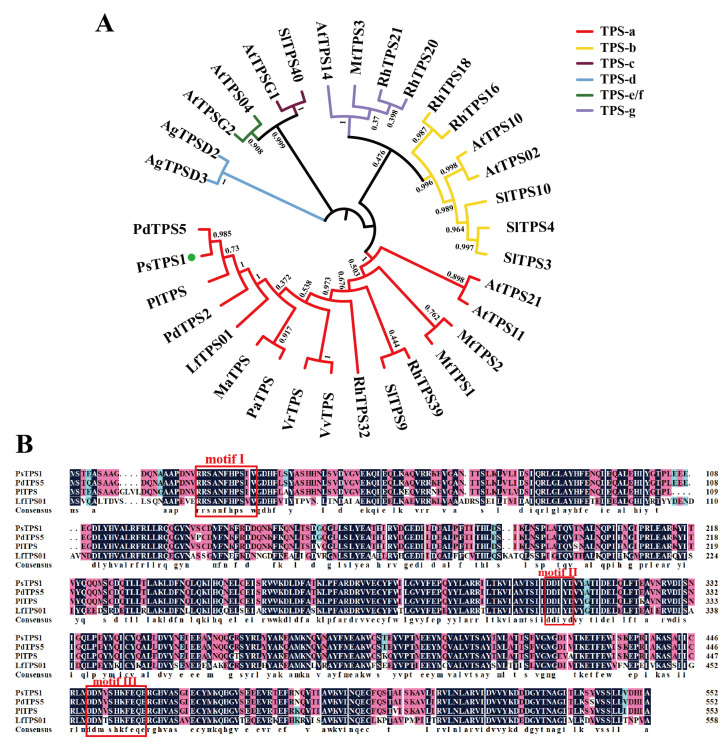
Phylogenetic tree and multiple sequence alignment of TPS proteins. (**A**) Phylogenetic tree of PsTPS1 and TPS proteins from other 12 plant species using the NJ method by MEGA X software V10.2.6. Bootstrap values are shown as a percentage of 1000 replicates. PsTPS1 is marked with a green dot. Clades of TPS-a, TPS-b, TPS-c, TPS-d, TPS-e/f, and TPS-g are highlighted with different color lines. At, *Arabidopsis thaliana*; Sl, *Solanum lycopersicum*; Mt, *Medicago truncatula*; Ag, *Abies grandis*; Lf, *Liquidambar formosana*; Ma, *Melia azedarach*; Pa, *Populus alba*; Pd, *Paeonia delavayi*; Pl, *Paeonia lactiflora*; Vr, *Vitis riparia*; Vv, *Vitis vinifera*; Rh, *Rosa hybrida*. (**B**) Alignment and analysis of PsTPS1 with TPS protein sequences of *P. delavayi*, *P. lactiflora*, and *L. formosana*. Motif I, R(R,P,Q)(X)_8_W; motif II, DDXXD; motif III, (N,D)DXX(S,T,G)XXXE (NSE/DTE). Accession information of TPS proteins of other 12 plant species is detailed in Supplemental Table S2.

Phylogenetic tree construction by downloading 32 representative TPS protein sequences of 9 other species from the TPS-a, TPS-b, TPS-c, TPS-d, TPS-e/f, and TPS-g subfamilies, respectively, showed that PsTPS1 clustered in the TPS-a subfamily (Figure 5A). And PsTPS1 also had a shorter branch length in the phylogenetic tree (Figure 5A), indicating that it differentiated later than other plants, the result that was the same as phylogenetic tree of PsHMGR1 in Figure 3A. In addition, PsTPS1 showed the closest homology to PdTPS (TPS of *P. delavayi*), which also belonged to the genus *Paeonia*, in agreement with the plant taxonomy, as well as clustering on the same branch with PlTPS of *P. lactiflora*, PdTPS2 of *P. delavayi*, and LfTPS01 of *Liquidambar formosana* (Figure 5A).

Based on the results of the tree, three TPSs were selected for multiple sequence alignment analysis. The results of multiple sequence alignment indicated that PsTPS1 had the conserved R(R,P,Q)(X)_8_W (motif I) at the N-termini that could catalyze the cyclization of substrates, as well as two typical DDXXD (motif II) and NSE/DTE (motif III) motifs at the C-termini that can be involved in the binding of divalent metal cations Mg^2+^ or Mn^2+^ (Figure 5B). Compared to the amino acid sequences of other species, the PsTPS1 protein had the highest identity to the TPS protein of *P. delavayi* (PdTPS) (99.10%), followed by the PlTPS (95.15%) and LfTPS01 (70.18%) (Figure 5B).

### 2.5. PsHMGR1 and PsTPS1 Genes Show Different Expression Patterns in Flower Development Stages and Different Tissues

**Figure 6:**
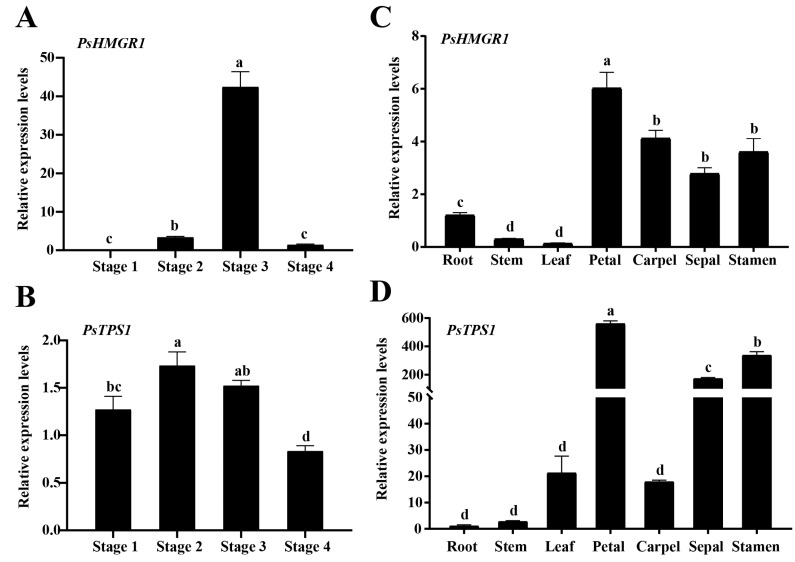
The expression patterns of *PsHMGR1* and *PsTPS1* genes. (**A**) Relative expression levels of *PsHMGR1* during four flower development stages. (**B**) Relative expression levels of *PsHMGR1* in different organs and tissues. (**C**) Relative expression levels of *PsTPS1* during four flower development stages. (**D**) Relative expression levels of *PsTPS1* in different organs and tissues. Data are presented as mean ± SE, *n* = 3. Different lowercase letters indicate significant differences at *p* < 0.05 level.

The expression patterns of *PsHMGR1* and *PsTPS1* were analyzed by qRT-PCR in *P. suffruticosasion* ‘Oukan’. The results indicated that both *PsHMGR1* and *PsTPS1* were expressed at every flower developmental stage and every organ and tissue (Figure 6). *PsHMGR1* expression levels increased from Stage 1 to Stage 3 and then decreased to Stage 4 (Figure 6A). The expression level of *PsTPS1* also showed a trend of increasing and then decreasing, with the highest expression level at Stage 2 (Figure 6B). The expression levels of the *PsHMGR1* and *PsTPS1* at different flower development stages were consistent with the release amount of floral fragrance volatile from ‘Oukan’ [42], demonstrating that they may play an important role in floral volatile biosynthesis.

In various organs and tissues, both *PsHMGR1* and *PsTPS1* were expressed at much higher levels in flowers than in roots, stems, and leaves, especially in petals and stamens (Figure 6C,D), suggesting that these two genes may play important roles in the formation of floral traits. The expression levels of *PsHMGR1* were also higher in carpels and sepals, which were not significantly different from those in stamens, and higher in roots than in stems and leaves (Figure 6C). Whereas *PsTPS1* was expressed at a significantly higher level in sepals than in carpels, it was not significantly differentially expressed in roots, stems, and leaves (Figure 6D).

### 2.6. Overexpression of PsHMGR1 and PsTPS1 in Tobacco Affects the Accumulation of Terpenoids

**Figure 7:**
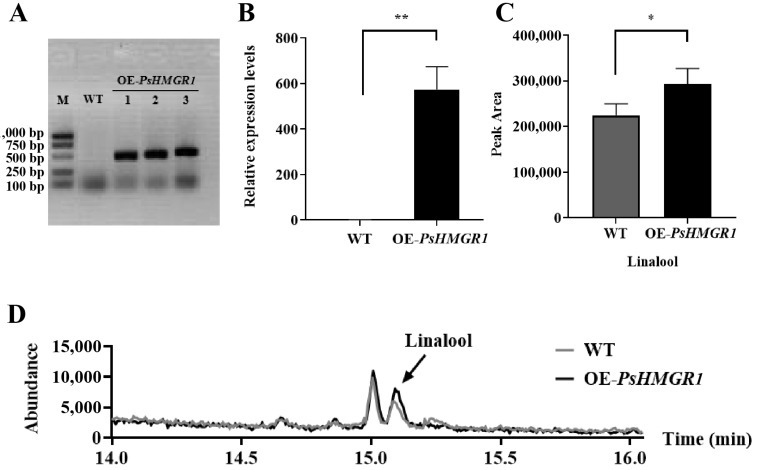
*PsHMGR1* gene relative expression levels and volatile emission amounts in transgenic plants and control. (**A**) PCR detection of transgenic plants and wild type (WT) tobaccos. M, marker; WT, wild type; OE-*PsHMGR1*, *PsHMGR1* transgenic tobacco lines. (**B**) The expression levels of *PsHMGR1* in transgenic lines and WT determined by qRT-PCR. The 18S gene was used as the endogenous control. (**C**) The emission amounts of linalool in *PsHMGR1* transgenic lines and WT. (**D**) The GC-MS detection of flower VOCs from *PsHMGR1* transgenic plants and wild-type tobacco. * *p* < 0.05, ** *p* < 0.01.

**Figure 8:**
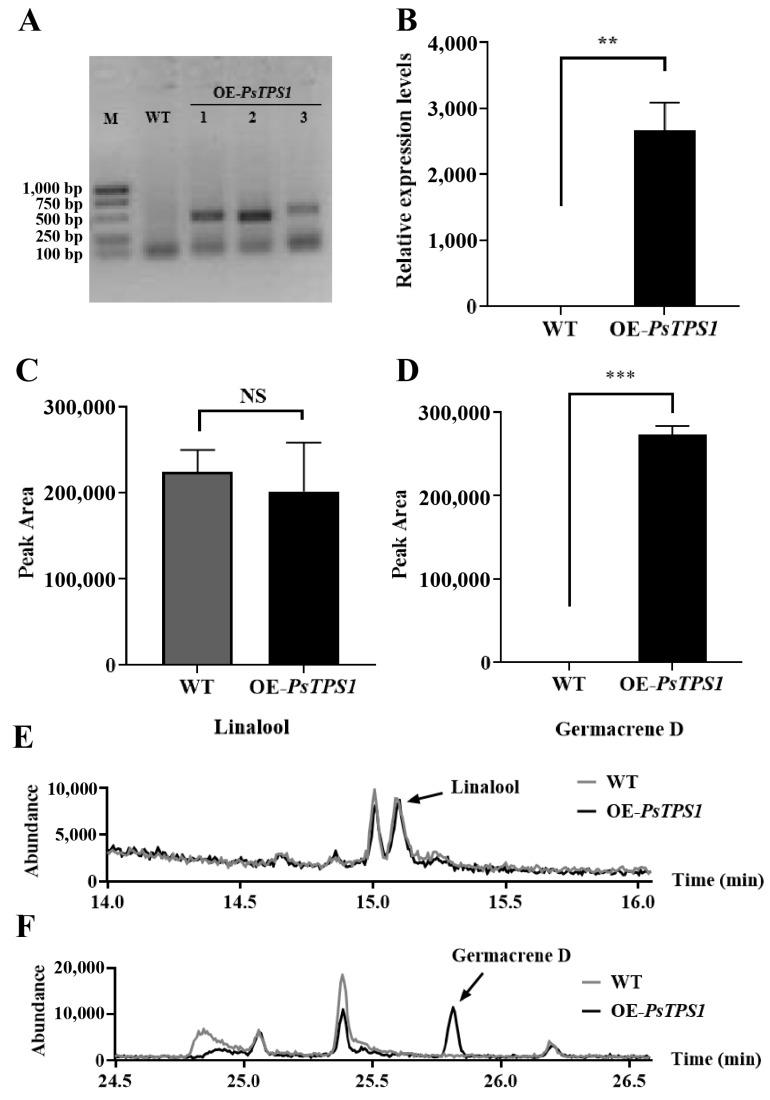
*PsTPS1* gene relative expression levels and volatile emission amounts in transgenic plants and control. (**A**) PCR detection of transgenic plants and wild-type tobaccos. M, marker; WT, wild type; OE-*PsTPS1*, *PsTPS1* transgenic tobacco lines. (**B**) The expression levels of *PsTPS1* in transgenic lines (OE-*PsTPS1*) and WT determined by qRT-PCR. (**C**,**D**) The emission amounts of linalool and germacrene D in *PsTPS1* transgenic lines and WT. (**E**,**F**) The GC-MS detection of flower VOCs from *PsTPS1* transgenic plants and wild-type tobacco. ** *p* < 0.01, *** *p* < 0.001, NS: non-significant.

Due to the lack of a functional validation system for *P. suffruticosa* ‘Oukan’, transgenic tobacco was selected for functional exploration of the *PsHMGR1* and *PsTPS1* genes, and the experimental procedure was displayed in Figure S3. Three positive transgenic lines harboring *PsHMGR1* (OE-*PsHMGR1*) (Figure 7A) and three lines harboring *PsTPS1* (OE-*PsTPS1*) (Figure 8A) were selected using PCR, and the wild-type (WT) tobacco lines were treated as controls. The expression levels of these two genes in transgenic lines were further confirmed by qRT-PCR. The results showed that the expression levels of both genes were significantly increased in the transgenic lines compared to the levels in the control (Figure 7B and Figure 8B). Detection of floral volatiles by headspace SPME-GC-MS revealed that overexpression of *PsHMGR1* resulted in significant changes in compound content in the transgenic lines. And the peak area of linalool (a monoterpene, which was the main floral volatile in ‘Oukan’) detected in the OE-*PsHMGR1* lines was 30.40% higher than that of the WT (Figure 7C,D).

For the transgenic lines of the *PsTPS1* gene, the content of volatile compounds changed considerably in the WT and transgenic lines of *PsTPS1* (OE-*PsTPS1*). The OE-*PsTPS1* lines showed a significant increase in germacrene D, a sesquiterpene volatile compound, which was not detected in the WT lines (Figure 8D,F). However, there was no significant difference in the linalool content detected (Figure 8C,E). And the complete GC-MS total ion chromatograms and substance identification table were shown in Figure S4 and Table S3.

## 3. Discussion

Floral fragrance is an important trait of tree peony, which not only mediates its pollination and environmental adaptation but also serves as a key ornamental evaluation index, determining its market value and potential for development and application. Compared with flower color and shape, the study of floral fragrance has started later. At present, the reports on tree peony floral scent mainly focus on the detection and analysis of volatile components, and only in the past three years molecular investigations gradually emerge [45,52,53,56,57]. However, the cloning and preliminary functional analyses of terpenoid synthesis genes for floral scent are focused on the variety of tree peony cultivar ‘High Noon’ [45,46,52,53,57]. As a leading cultivar from the Japanese cultivar-group, the tree peony cultivar ‘Oukan’, with its strong floral scent, unique flower shape, golden color, high adaptability, and ease of cultivation and management, is not only highly attractive but also has great potential in breeding, fragrance products, and food industry applications. Tree peony cultivar ‘Oukan’ has been reported to have a strong floral fragrance dominated by terpenoids, reaching more than 80% [42]. While there is a lack of investigation on the molecular synthesis mechanism of terpenoids in its floral fragrance. Therefore, in this study, we used ‘Oukan’ as the material and screened two terpene synthesis pathway genes, *PsHMGR1* and *PsTPS1*, to carry out further work on the molecular level research.

HMGR is widely distributed in various types of organisms. The characterization and amino acid sequence analysis of a large number of HMGR genes reveals that HMGR is divided into two major classes: class I and class II. Class I is found in eukaryotes with highly conserved catalytic domains and a transmembrane region at the N-terminal associated with the degradation of HMGR molecules, while class II is first discovered in prokaryotes and usually have no transmembrane region [25,26]. In this study, analysis of the amino acid sequence encoded by the *PsHMGR1* gene of ‘Oukan’ showed that the putative PsHMGR1 protein contained structural features common to most plant HMGRs: it was attributed to the HMG-CoA_reductase superfamily member and contained a typical HMG-CoA_reductase_class I structural domain; highly conserved at the C-terminus with large sequence variation at the N-terminus; two transmembrane structural domains; and four highly conserved motifs, i.e., two HMG-CoA-binding motifs and two NADP(H)-binding motifs [24,25,26]. We found that the amino acids of the second HMG-CoA-binding site and the two NADP(H)-binding sites of PsHMGR1 were identical to those of other plants, whereas the first HMG-CoA-binding site varied somewhat among different plant HMGRs, and the differences in this site might affect the recognition and binding ability to substrates [25]. Phylogenetic tree results showed that PsHMGR1 had high homology with DtHMGR and PtHMGR proteins, and *D. turbinata* also possessed volatile floral fragrance, as well as producing significantly higher terpene content than the control in the *PtHMGR* transgenic poplar lines [32]. So it was hypothesized that *PsHMGR1* was involved in the biosynthesis of floral fragrance volatiles.

Further examination of the expression level of *PsHMGR1* in different organs of ‘Oukan’ revealed that its expression level in flower was significantly higher than that in other organs. The changes in the expression of *PsHMGR1* during the four flower developmental stages showed a consistent trend with the changes in floral fragrance release. The study reported that the expression levels of plant *HMGR* genes in different tissues were closely correlated with the accumulation of metabolites, which further illustrated the potential of *PsHMGR1* in floral scent biosynthesis [24]. In *Jasminum sambac*, the expression level of *JsHMGR* also shows a trend of increasing and then decreasing along with different flowering stages, which is basically consistent with the trend of changes in the floral fragrance volatiles content [58], which is in agreement with *PsHMGR1*. Currently, *HMGR* genes have been isolated and functionally characterized mainly in medicinal plants, such as *A. annua* [59], *Panax ginseng* [29], *Pseudostellaria heterophylla* [26], *Catharanthus roseus* [60], and *Lithospermum erythrorhizon* [24]. HMGR enzyme activity as well as total triterpene content is significantly higher in tobacco plants transgenic for *PhHMGR* than in control plants [26]. *LerHMGR1* and *LerHMGR2* from *L. erythrorhizon* have been cloned and demonstrated in vitro to catalyze the conversion of HMG-CoA to MVA, which is thought to play a key rate-determining role in the biosynthesis of shikonin [24]. Transgenesis of the *hmgr* gene from *C. roseus* into *A. annua* produces 38.9% higher artemisinin than the control and increased HMGR enzyme activity [60]. However, little has been reported on the functional resolution of the *HMGR* gene in ornamental flowers. A mature genetic transformation system has not yet been established in tree peony. In this study, *PsHMGR1* was transformed into tobacco, and linalool release amount was detected to be significantly higher than the control. Our work complements the functional characterization of *HMGR* genes in ornamental plants. The expression levels of *JsHMGR* in *J. sambac* at different stages of flower development and at different times after bud excision are basically in agreement with the trend of linalool release [58], which further corroborates our results.

As a key enzyme in the last step of terpene synthesis, TPS has a direct role in determining the type of terpene compounds synthesized. Although its sequence is somewhat conserved, TPS proteins are diverse during evolution and in different plants [4,10,61]. PsTPS1 isolated in this study possessed the typical conserved structural domain Terpene_cyclase_plant_C1, which was usually contained in TPS family members; had the highly conserved DDXXD and NSE/DTE motifs, which were considered to be active sites necessary for binding divalent metal cations to catalyze the biosynthesis of terpenes; and was also found to have a typical R(R,P,Q)(X)_8_W motif at the N-terminus responsible for catalyzing the monoterpenes cyclization [8,18,61]. Typically, members of the TPS-a subfamily synthesize mainly sesquiterpenes [18,62]. In *F. hybrida*, TPS-a subfamily members FhTPS6, FhTPS7, and FhTPS8 all synthesize the sesquiterpenes [18], and five TPS-a subfamily members in *Rosa hybrida*, RhTPS47, RhTPS26, RhTPS30, RhTPS32, and RhTPS32S, are also identified as sesquiterpene synthases [61]. The results of phylogenetic tree analysis in this study showed that PsTPS1 was clustered into the TPS-a clade, so it was hypothesized that PsTPS1 might be involved in sesquiterpene synthesis. PdTPS5, PdTPS2, and RhTPS32, which are more homologous to PsTPS1 in the tree, have been reported to produce sesquiterpene germacrene D in vitro [52,61]. PaTPS, VrTPS, and VvTPS, which were clustered on the same branch as PsTPS1, were all annotated as germacrene D synthases in NCBI, further speculating that PsTPS1 had the potential to synthesize the sesquiterpene germacrene D. Subsequently, qRT-PCR revealed that the expression levels of *PsTPS1* in ‘Oukan’ at different flower developmental stages were consistent with the trend of floral scent release, and the highest expression levels were found in the petal. Further, germacrene D, which was not present in the WT lines, was detected in *PsTPS1* transgenic tobacco flowers, and this result validated our previous analyses. Thus, PsTPS1 could be considered a terpene synthase gene that catalyzes germacrene D synthesis exclusively, with potential in the future molecular breeding of tree peony floral fragrances.

In this study, *PsHMGR1* and *PsTPS1* genes potentially affecting the biosynthesis of floral fragrance terpenoids in tree peony cultivar ‘Oukan’, were cloned and bioinformatically analyzed based on the results of transcriptome analysis, and the functions of *PsHMGR1* and *PsTPS1* were further characterized by gene expression level assays as well as transgenic approaches. Notably, significantly higher linalool release amount was detected in the flowers of the *PsHMGR1* transgenic lines than in WT; germacrene D, which was absent in WT, was detected in the flowers of the *PsTPS1* transgenic lines, but there was no differential change in linalool release amount. This suggested that these two genes played an important role in the synthesis of floral terpene volatiles, and it was hypothesized that there was a relationship between the MVA and MEP pathways. Our results provide new insight into the study of floral fragrances of tree peony ‘Oukan’ at the molecular level.

## 4. Materials and Methods

### 4.1. Plant Materials

*P. suffruticosa* ‘Oukan’ was grown in Beijing University of Agriculture (Beijing, China. 116°3′14″ E, 40°0′95″ N). It was divided into four different flower developmental stages, namely the bud brusting stage (stage 1), the initial flowering stage (stage 2), the full blooming stage (stage 3), and the flower withering stage (stage 4) (Figure 1A). And the roots, stems, leaves, petals, stamens, carpels, and sepals samples from stage 3 and petals from the four stages were collected. For total RNA extraction, samples were immediately frozen in liquid nitrogen and stored at −80 °C for further experiments. Tobacco plants for transformation were grown in an artificial climate room under standard conditions (16 h of light/8 h of dark, 25 ± 2 °C).

### 4.2. RNA Extraction, cDNA Synthesis, and Expression Analyses (qRT-PCR)

Total RNA was extracted from petals of different stages, organs, and tissues using an EASY Spin Plant RNA Extraction Kit (Aidlab, Beijing, China). And the integrity and concentration of RNA were detected by 1.2% agarose gel electrophoresis and nucleic acid detectors (Implen Nanophotometer P330, Munich, Germany), respectively. cDNA was synthesized in a final reaction volume of 20 µL, referring to the *Evo M-MLV* Reverse Transcription (Accurate Biotechnology (Hunan) Co., Ltd., Changsha, AG11605, China).

Specific primers (Table 1) were designed based on transcriptome sequencing data [42]. The expression levels of the genes were detected by qRT-PCR using cDNA as a template and referring to the SYBR Green Premix *Pro Taq* HS qPCR Tracking Kit (Accurate Biotechnology (Hunan) Co., Ltd., Changsha, AG11735, China). There were three independent biological replicates and three technical replicates of each biological replicate. *Actin* (Table 1) was used as an internal reference gene. The relative expression levels of the genes were calculated and analyzed using the 2^−ΔΔCT^ method. Data were statistically analyzed using ONE-WAY ANOVA in GraphPad Prism V8.4.2.

### 4.3. Gene Cloning and Sequence Analysis

Candidate genes were selected from DEGs analysis on the terpene metabolic pathway of *P. suffruticosa* transcriptome database [42]. The cDNAs were synthesized from the total RNA of the petals in Stage 3 of *P. suffruticosa* ‘Huangguan’ as the templates, and PCR reactions were referenced to *ApexHF* HS DNA polymerase FS Master Mix (Accurate Biotechnology, AG12206, China). According to tree poney transcriptome data, *PsHMGR1* and *PsTPS1* genes were cloned using the RACE-PCR approach. The PCR product was cloned into the pClone007 vector (Beijing Tsingke Biotech Co., Ltd., TSV-007VS, Beijing, China) and transformed into *E.coil* DH5α for sequencing confirmation. Primers were listed in Table 1.

The physicochemical properties, secondary structure characterization, 3D structure model building, transmembrane domains, hydrophilic or hydrophobicity analysis, protein signal peptide sequences, and protein conserved domains of PsHMGR1 and PsTPS1 proteins were performed using the Expasy ProtParam tool, SOPMA, SWISS-MODEL server, TMHMM2.0, Expasy ProtScale, SignalP 6.0, and NCBI Conserved Domains V3.21 software, respectively.

### 4.4. Phylogenetic Trees Construction and Multiple Sequence Alignment

For constructing the phylogenetic tree of PsHMGR1, firstly, HMGR proteins with high homology to PsHMGR1 were found by NCBI Blastp from ten species of plants, namely *D. turbinata*, *T. wilfordii*, *Ricinus communis*, *P. trichocarpa*, *Nelumbo nucifera*, *P. euphratica*, *Mangifera indica*, *P. alba*, *Pistacia vera*, and *Jatropha curcas*. Subsequently, 27 HMGR protein sequences from 25 representative species were downloaded based on reported HMGR studies from 3 animals (*Homo sapiens*, *Rattus norvegicus* and *Drosophila albomicans*), 3 fungi (*Saccharomyces cerevisiae*, *Ganoderma lucidum* and *Lachnellula hyalina*), 3 bacteria (*Zobellia galactanivorans*, *Streptomyces malaysiensis* and *Brevibacterium linens*), 2 lower plants (*Chlorokybus atmophyticus* and *Mesostigma viride* from the Chlorophyta), 3 Pteridophyta (*Alsophila spinulosa*, *Ceratopteris richardii* and *Adiantum capillus*), 2 Bryophytes (*Physcomitrella patens* and *Marchantia polymorpha*), 2 Gymnosperms (*Pinus tabuliformis* and *Ginkgo biloba*), and 7 Angiosperms (1 basal angiosperm *Amborella trichopoda*, 2 monocots *Oryza sativa* and *Zea mays*, and 4 eudicots *Olea europaea*, *Vitis vinifera*, *P. trichocarpa* and *Arabidopsis thaliana*) [24,26]. Information on these HMGR proteins is shown in Table S1. Finally, the phylogenetic tree was constructed by ‘One Step Build a ML Tree’ in TBtools V2.136 software with a bootstrap value of 5000 [63].

Homologous proteins of PsTPS1 were found and downloaded in NCBI Blastp, including *P. delavayi*, *P. lactiflora*, and *L. formosana*. Also refer to the papers [18,35,61] to download TPS proteins from TPS-a, TPS-b, TPS-c, TPS-d, TPS-e/f, and TPS-g subfamilies, including *A. thaliana*, *Solanum lycopersicum*, *Medicago truncatula*, *Abies grandis*, *Melia azedarach*, *P. alba*, *Vitis riparia*, *V. vinifera*, and *Rosa hybrida*. Information on the TPS proteins is shown in Table S2. Then, the phylogenetic tree was constructed employing the MEGA-X V10.2.6 software with the neighbor-joining (NJ) method, and a bootstrap value of 1000 was used to verify the phylogenetic tree. Finally, the generated tree was visualized by online software iTOL.

Open reading frame (ORF) analysis and multiple sequence alignment of PsHMGR1, PsTPS1, and proteins from other species were carried out with DNAMAN V9.0 software. Motifs were analyzed with reference to the reported paper [8,24,26,64].

### 4.5. Overexpression PsHMGR1 and PsTPS1 in Tobacco and qRT-PCR Detection

The ORF sequence of *PsHMGR1* without terminator and the enzymatic sites *Bma*HΙ and *Xba*Ι were used for primer design (Table 1), and the pCAMBIA1301-*PsHMGR1* functional vector was constructed by homologous recombination. At the same time, the pCAMBIA1301-*PsTPS1* recombinant vector was constructed by designing primers (Table 1) according to the enzymatic sites *Eco*RΙ and *Xba*Ι. The two recombinant vectors pCAMBIA1301-*PsHMGR1* and pCAMBIA1301-*PsTPS1* were transferred into GV3101 competent cells from *Agrobacterium tumefaciens* (Weidi Biotechnology Co., Shanghai, China), respectively. The colonies were incubated at 28 °C for 2 days and then verified by PCR. The positive clones were amplified with shaking, and the bacteria were collected by centrifugation and resuspended with the infiltration solution to OD_600_ = 0.2.

Small pieces of sterile tobacco leaves after 3 days of pre-culturing were immersed in the infiltration solution for 10 min, taken out and dried on filter paper, and then inoculated on co-culture medium for dark culture. After 2 days, they were transferred to induction medium to induce healing tissues. Subsequently, the healing tissues that met the requirements were selected for screening, differentiation, rooting, and flowering.

Total genomic DNA was extracted from young leaves of tobacco resistant plants with reference to the Hi-DNAsecure Plant kit (Beijing Tiangen Biochemical Technology Co., Ltd., DP350, Beijing, China). The DNA was used as a template for PCR amplification using hyg(501)-F/R as primers to detect the positive transgenic tobacco lines. For positive lines, at least 0.5 g of petals from the transgenic tobacco flowers were pooled and RNA extracted by referring to the *TransZol* Up Plus RNA Kit (TransGen Biotech, ER501-01, Beijing, China). And qRT-PCR was performed following the steps described above using 18S as the internal reference gene. The sequences of all the gene-specific primers were listed in Table 1. Statistical analysis was performed using the *t*-test method.

### 4.6. Floral Scent Collection, Determination and Analysis

Floral scent compounds were extracted by headspace SPEM using a 50/30 μm divinylbenzene/carboxen/polydimethylsiloxane (DVB/CAR/PDMS) coated fiber attached to a manual SPME holder (Supelco, Bellefonte, PA, USA). The fiber was conditioned in the GC injection port for 30 min at 250 °C before the first volatile collection. For positive lines, 0.5 g of tobacco fresh flower of full blooming were put into a 20 mL clear glass vial enclosed with a cap. The sample was equilibrated for 3 h at 25 °C, and collection was done at 50 °C for 35 min using the extractor inserted into the upper side of the vial.

The collected aromatic compounds were subsequently transferred to an Agilent 5975C GC-MS instrument (Agilent Technologies, Santa Clara, CA, USA) and desorbed for 3 min at a time. The chromatography program started at 40 °C for 2 min, and the temperature was increased to 200 °C at a rate of 5 °C/min and held at this temperature for 6 min; the inlet temperature was set at 200 °C, total flow was 27.5 mL/min, and the split ratio was 20:1.

The composition of the volatile compounds was identified using the NIST14 database through an MSD Productivity ChemStation (Agilent Technologies, Santa Clara, CA, USA). The peak areas of these compounds were counted.

### 4.7. Statistical Analysis

All experiments were conducted in triplicate. Excel and GraphPad Prism 8.4.2 software were used for data organization, significance analysis, and visualization.

## 5. Conclusions

In this study, two key genes, *PsTPS1* and *PsHMGR1*, associated with floral fragrance terpene synthesis, were cloned firstly in *P. suffruticosa* ‘Oukan’. Protein multiple sequence alignment and phylogenetic tree analysis revealed that PsHMGR1 belongs to the HMG-CoA_reductase A superfamily, and PsTPS1 belongs to the TPS-a subfamily. During different flower development stages, The expression levels of *PsHMGR1* and *PsTPS1* increased and then decreased, with the highest expression of *PsHMGR1* in stage 3 and *PsTPS1* in stage 2. In different organs and tissues, the expression levels of *PsHMGR1* and *PsTPS1* were significantly higher in the organ of the flower than in the root, stem, and leaf and were highly expressed in the petal and stamen. Subsequently, the PsHMGR1 and PsTPS1 genes were transgenic into tobacco, respectively. After obtaining resistant plants, three transgenic strains were identified by DNA and RNA analysis. Flower fragrance determination of the transgenic tobacco lines showed significantly higher terpene volatile content compared to the WT lines. The *PsHMGR1* transgenic lines produced higher levels of linalool compared to the WT, while the *PsTPS1* transgenic lines produced new germacrene D. Our study contributes new novel perspectives on the molecular mechanisms of biosynthesis of floral fragrance terpenoid volatiles in ‘Oukan’. The findings provide genetic support for terpenoid synthesis in tree peony and are expected to be used for molecular design breeding for its floral fragrance trait.

## Figures and Tables

**Table 1:** Primers used in the experiments.

Primer Name	Primer Sequence (5′-3′)	Utilization
*PsHMGR*-F	ATGGACGTTCGCCGACGACCA	Gene cloning
*PsHMGR*-R	TTAAGAAGCAACTTTGGATACG	
*PsTPS1*-F	ATGTCTACTGAAGCTTCC	
*PsTPS1*-R	TCATATTGCAATATGATCAAC	
*qPsHMGR*-F	CGTCAAAGTGAAGCGAGTAATG	qRT-PCR
*qPsHMGR*-R	AAATGCAGGCAGGTTCGTTC	
*qPsTPS1*-F	TGGGCTCTCGGTTTTCTTCA	
*qPsTPS*-1R	TTCGCTTTAGGACTTCGGGT	
*18S*-F	CGCTCTGGATACATTAGCATGG	
*18S*-R	CGTTGGATGAAGAACCCCCA	
*Actin*-F	CGGTGTCTGGATTGGAGGGTCA	
*Actin-R*	TTCGCTTTAGGACTTCGGGT	
*pCAMBIA1301*-*PsHMGR*-F	TATGACCATGATTACGAATTCATGGACGTTCGCCGACG	Vector construction
*pCAMBIA1301*-*PsHMGR*-R	TGCCTGCAGGTCGACTCTAGAAGAAGCAACTTTGGATACGTCTTTG	
*pCAMBIA1301*-*PsTPS1*-F	GGAATTCCATGTCTACTGAAGCTTCC	
*pCAMBIA1301*-*PsTPS1*-R	CGGGATCCCGTCATATTGCAATATGATCAAC	
hyg(501)-F	GAGCATATACGCCCGGAGTC	Positive PCR testing
hyg(501)-R	CAAGACCTGCCTGAAACCGA	

## Data Availability

All data generated or analyzed during this study are included in this published article. Data are contained within the article and Supplementary Materials.

## Supplementary Materials

The following supporting information can be downloaded at: https://www.mdpi.com/article/10.3390/ijms252212247/s1.
