# Supplementary material for: Functional Analysis of PsHMGR1 and PsTPS1 Related to Floral Terpenoids Biosynthesis in Tree Peony
Source: Int J Mol Sci. 2024 Nov 14;25(22):12247. doi: 10.3390/ijms252212247 (PMC11594739; doi:10.3390/ijms252212247)
Supplement: Supplementary file 1 [file ijms-25-12247-s001.zip › ijms-3260161-supplementary.pdf]

```
1       ATGGACGTTCGCCGACGACCACCAAAACCTCCTCGTCCGGGTACCGACACACATGGCGGATCTTCCAATCACCACCACCTCCACCAGAAA
1        M  D  V  R  R  R  P  P  K  P  P  R  P  G  T  D  T  H  G  G  S  S  N  H  H  H  L  H  Q  K
91      AAGTCATCATCTTCCGTCGGTAATCGCTCGCAATCTCCTGCCCCAAAAGCATCGGATGCCCTTCCACTCCCACTATACCTAACCAACACT
31       K  S  S  S  S  V  G  N  R  S  Q  S  P  A  P  K  A  S  D  A  L  P  L  P  L  Y  L  T  N  T
181     ATATTCTTCACCCTCTTCTTCTCGGTGGCTTACTACCTCCTCCACCGGTGGCGCGATAAGATCCGTACGTCTACGCCCCTCCACGTCGTT
61       I  F  F  T  L  F  F  S  V  A  Y  Y  L  L  H  R  W  R  D  K  I  R  T  S  T  P  L  H  V  V
271     ACCCTATCCGAAATTGCCGCCATTGTCTCTCTAATTGCGTCCTTCATCTATCTCCTCGGTTTCTTCGGTATCGATTTCGTTCAATCTTTC
91       T  L  S  E  I  A  A  I  V  S  L  I  A  S  F  I  Y  L  L  G  F  F  G  I  D  F  V  Q  S  F
361     ATCGCCCGTGCCTCACACGATGCCTGGGATGTTGAAGACGAAACAGAACGCTTATTCATCGAAGATCGCCATCTTGGACCGGTAGTTCCT
121      I  A  R  A  S  H  D  A  W  D  V  E  D  E  T  E  R  L  F  I  E  D  R  H  L  G  P  V  V  P
451     CCAGTCGCCGCCATTGCTCCGCTTCCCAAATTGGTTAATTCGACACCGGACATTATAATTCCGCCGTCCGAAGAAGACGAGGAGATCATC
151      P  V  A  A  I  A  P  L  P  K  L  V  N  S  T  P  D  I  I  I  P  P  S  E  E  D  E  E  I  I
541     AGATCGGTAGTTGCGGGCGAGATTCCCTCATACTCACTCGAATCAAAGCTCGGAGACTGTAAGCGTGCGGCAGTGATTCGCCGTGAAGCT
181      R  S  V  V  A  G  E  I  P  S  Y  S  L  E  S  K  L  G  D  C  K  R  A  A  V  I  R  R  E  A
631     CTCCAGAGAATTACTGGTAGGTCACTTCAAGGGTTACCGTTAGACGGATTCGATTATGAGTCAGTTTTAGGTCAGTGCTGTGAGATGCCA
211      L  Q  R  I  T  G  R  S  L  Q  G  L  P  L  D  G  F  D  Y  E  S  V  L  G  Q  C  C  E  M  P
721     GTTGGGTATATTCAGATTCCGGTAGGGATTGCGGGGCCATTGTTGCTCGATGGGTTAGAGTATTCGGTGCCGATGGCGACAACCGAAGGG
241      V  G  Y  I  Q  I  P  V  G  I  A  G  P  L  L  L  D  G  L  E  Y  S  V  P  M  A  T  T  E  G
811     TGTTTGGTGGCTAGTACAAATAGGGGTTGTAAGGCAATTTATCTTTCCGGCGGGGCAGAGAGTACGTTGTTGAGAGATGCGATGACTAGA
271      C  L  V  A  S  T  N  R  G  C  K  A  I  Y  L  S  G  G  A  E  S  T  L  L  R  D  A  M  T  R
901     GCACCGGTTGTGAGATTTGGATCGGCGAAGAGGGCTTCTGAGTTGAAATTCTTCTTGGAGGACCCCGTCAATTTCGACACTTTAGCCGTT
301      A  P  V  V  R  F  G  S  A  K  R  A  S  E  L  K  F  F  L  E  D  P  V  N  F  D  T  L  A  V
991     GTTTTTAATAGGTCAAGTAGATTTGGAAGGCTTCAAAATATTAAATGCTCTATTGCTGGAAAAATCTTTACATGAGATTTAGCTGCAGT
331      V  F  N  R  S  S  R  F  G  R  L  Q  N  I  K  C  S  I  A  G  K  N  L  Y  M  R  F  S  C  S
1081    ACAGGTGATGCAATGGGGATGAACATGGTGTCTAAAGGGGTTCAAAACGTTTTAGATTTCCTTCAGAGTGATTTTCCAGACATGGATGTC
361      T  G  D  A  M  G  M  N  M  V  S  K  G  V  Q  N  V  L  D  F  L  Q  S  D  F  P  D  M  D  V
1171    ATAGGCATCTCTGGGAATTTTTGTTCGGACAAGAAACCGGCTGCAGTAAACTGGATCGAAGGGCGTGGGAAATCAGTTGTTTGTGAGGCA
391      I  G  I  S  G  N  F  C  S  D  K  K  P  A  A  V  N  W  I  E  G  R  G  K  S  V  V  C  E  A
1261    ATAATTAAGGAAGAAGTAGTGAAGAAGGTATTGAAAACTGATGTAGCTTCTTTGGTAGAGCTCAACATGCTAAAGAACCTAGCTGGTTCT
421      I  I  K  E  E  V  V  K  K  V  L  K  T  D  V  A  S  L  V  E  L  N  M  L  K  N  L  A  G  S
1351    GCCATTGCTGGTGCGCTTGGTGGGTTCAATGCCCATGCCAGTAATATCGTGTCTGCGATATTTATCGCCACCGGCCAAGATCCAGCCCAA
451      A  I  A  G  A  L  G  G  F  N  A  H  A  S  N  I  V  S  A  I  F  I  A  T  G  Q  D  P  A  Q
1441    AACATTGAGAGTTCTCATTGTATTACCATGATGGAACCTGTTAATGAGGGGAAAGATCTCCACATCTCAGTTACCATGCCTTCCATTGAG
481      N  I  E  S  S  H  C  I  T  M  M  E  P  V  N  E  G  K  D  L  H  I  S  V  T  M  P  S  I  E
1531    GTGGGTACAGTTGGTGGTGGAACTCAACTTGCATCTCAGTCAGCTTGTCTGAACTTGCTGGGTGTGAAAGGTGCGAGCATAGAGTCACCA
511      V  G  T  V  G  G  G  T  Q  L  A  S  Q  S  A  C  L  N  L  L  G  V  K  G  A  S  I  E  S  P
1621    GGGTCCAACTCAAGGGTGTTGGCTAGTATCATATCAGGCTCTGTTTTGGCAGGGGAGCTGTCGTTGATGTCTGCCATTGCATCTGGACAG
541      G  S  N  S  R  V  L  A  S  I  I  S  G  S  V  L  A  G  E  L  S  L  M  S  A  I  A  S  G  Q
1711    CTTGTTAAGAGTCACATGAAATATAACCGATCCAGCAAAGACGTATCCAAAGTTGCTTCTTAA
571      L  V  K  S  H  M  K  Y  N  R  S  S  K  D  V  S  K  V  A  S  *
```

Figure S1. *PsHMGR* gene sequence and encoded amino acid sequence of tree peony cultivar 'Oukan'.

```
1       ATGTCTACTGAAGCTTCCGCTGCCGGTGATCAAAATGCTGCTGCGCCGGATAATGTTCGTCGGTCTGCCAATTTTCATCCTTCCATTTGG
1        M  S  T  E  A  S  A  A  G  D  Q  N  A  A  A  P  D  N  V  R  R  S  A  N  F  H  P  S  I  W
91      GGTGATCATTTCTTGTCATACGCTTCTCATCATAACCTGTCGGTTGATGTTGGTGTGGAAAAACAAATTGAACAACTGAAAGCACAAGTA
31       G  D  H  F  L  S  Y  A  S  H  H  N  L  S  V  D  V  G  V  E  K  Q  I  E  Q  L  K  A  Q  V
181     AGGAGAAAATTTGTTGGTGCTAATACCACCTCGCTTAAGTTGGTCTTGATCGACTCGATTCAACGGTTAGGCTTGGCTTATCATTTTGAA
61       R  R  K  F  V  G  A  N  T  T  S  L  K  L  V  L  I  D  S  I  Q  R  L  G  L  A  Y  H  F  E
271     AACCAGATCGAACAAGCATTGGAGCATATTTATGGTACCCCTCTTGAGGAGGAGGAGGGTGACCTCTACCATGTTGCCCTCCGCTTTAGG
91       N  Q  I  E  Q  A  L  E  H  I  Y  G  T  P  L  E  E  E  E  G  D  L  Y  H  V  A  L  R  F  R
361     CTTCTCAGACAACAGGGCTACAATGTTTCATGTGACATGTTCAACAAGTTTAGGGATGACCAAAACAAGTTCAAACAAAATTTAACTAGC
121      L  L  R  Q  Q  G  Y  N  V  S  C  D  M  F  N  K  F  R  D  D  Q  N  K  F  K  Q  N  L  T  S
451     GATGGCCAGGGCCTTCTGAGCTTGTATGAGGCTACGCACATTCGAGTGGATGGAGAAGACATTCTAGATGAAGCCCTTCCTTTCACTATC
151      D  G  Q  G  L  L  S  L  Y  E  A  T  H  I  R  V  D  G  E  D  I  L  D  E  A  L  P  F  T  I
541     ACACACCTTGACTCCATAAAATTAAATTCACCTCTTGCAACACAAGTAACTAATGCTTTAAACCAGCCCATCCACATGGGCATACCTAGG
181      T  H  L  D  S  I  K  L  N  S  P  L  A  T  Q  V  T  N  A  L  N  Q  P  I  H  M  G  I  P  R
631     TTGGAGGCAAGAAAATACATCACTGTATATCAACAACAAATGTCATGTGATCAAACTCTCCTCACACTCGCAAAGTTAGATTTCAACCAA
211      L  E  A  R  K  Y  I  T  V  Y  Q  Q  Q  M  S  C  D  Q  T  L  L  T  L  A  K  L  D  F  N  Q
721     TTACAGAAAATTCACCAAAACGAGTTATGTGAAATTTCAAGGTGGTGGAAAGATTTGGACTTTGCAACAAAGCTACCTTTTGCTAGAGAT
241      L  Q  K  I  H  Q  N  E  L  C  E  I  S  R  W  W  K  D  L  D  F  A  T  K  L  P  F  A  R  D
811     AGGGTGGTGGAGTGCTATTTTTGGATTTTGGGAGTGTACTTCGAGCCCCAGTATTACCTAGCTAGAAGGATACTTACTAAAGTTATTGCC
271      R  V  V  E  C  Y  F  W  I  L  G  V  Y  F  E  P  Q  Y  Y  L  A  R  R  I  L  T  K  V  I  A
901     ATGACCTCCATTATCGATGATATATATGATGTGTATGCCACAATTGATGAACTTCAACTATTCACAGAAGCAGTTAACAGGTGGGATATA
301      M  T  S  I  I  D  D  I  Y  D  V  Y  A  T  I  D  E  L  Q  L  F  T  E  A  V  N  R  W  D  I
991     AGCAACATTGGTCAACTCCCAGAATACATGCAAATATGTTATCAAGCCCTCTTAGATGTTTATAATGAAATAGAGGAAGCAATGAACCAA
331      S  N  I  G  Q  L  P  E  Y  M  Q  I  C  Y  Q  A  L  L  D  V  Y  N  E  I  E  E  A  M  N  Q
1081    CAAGGAAGATCTTACCGCCTTTATTATGCGAAAGAAGCTATGAAAAATCAAGTGAACGCCTATTTTATGGAAGCCAAATGGTGTAGCACA
361      Q  G  R  S  Y  R  L  Y  Y  A  K  E  A  M  K  N  Q  V  N  A  Y  F  M  E  A  K  W  C  S  T
1171    GAATATGTGCCCACAATGGAGGAGTATATGCAGGTTGCGTTAGTCACCTCAGCTTACACCATGCTCGCAACAACATCTTTTGTTGGAATG
391      E  Y  V  P  T  M  E  E  Y  M  Q  V  A  L  V  T  S  A  Y  T  M  L  A  T  T  S  F  V  G  M
1261    GGAGACATTGTAACCAAGGAGACATTTGAATGGATCTCAAAAGAACCTAGAATTGCAAAGGCTTCTGCAATAATTTGTAGGCTCATGGAC
421      G  D  I  V  T  K  E  T  F  E  W  I  S  K  E  P  R  I  A  K  A  S  A  I  I  C  R  L  M  D
1351    GATATGGTGTCCCACAAGTTTGAGCAAGAGAGAGGGCATGTGGCATCTGGTATTGAATGTTACATGAAGCAACATGGTGTTTCAGAGGAG
451      D  M  V  S  H  K  F  E  Q  E  R  G  H  V  A  S  G  I  E  C  Y  M  K  Q  H  G  V  S  E  E
1441    GAAGTGCGCACTGAGTTTCGTAATCAAGTCACCATTGCATGGAAGGTCATTAATCAAGAATGCTTTCAATCAACGGCTATTTCCAAGGCC
481      E  V  R  T  E  F  R  N  Q  V  T  I  A  W  K  V  I  N  Q  E  C  F  Q  S  T  A  I  S  K  A
1531    GTCCTTATCCGCGTTCTTAACCTTGCTCGAGTGATTGATGTCGTGTACAAGGATGACGATGGTTACACAAATGCAGGGATCACTTTGAAG
511      V  L  I  R  V  L  N  L  A  R  V  I  D  V  V  Y  K  D  D  D  G  Y  T  N  A  G  I  T  L  K
1621    AGTTATGTGTCCTCATTACTCGTTGATCATATTGCAATATGA
541      S  Y  V  S  S  L  L  V  D  H  I  A  I  *
```

Figure S2. *PsTPS1* gene sequence and encoded amino acid sequence of tree peony cultivar 'Oukan'.

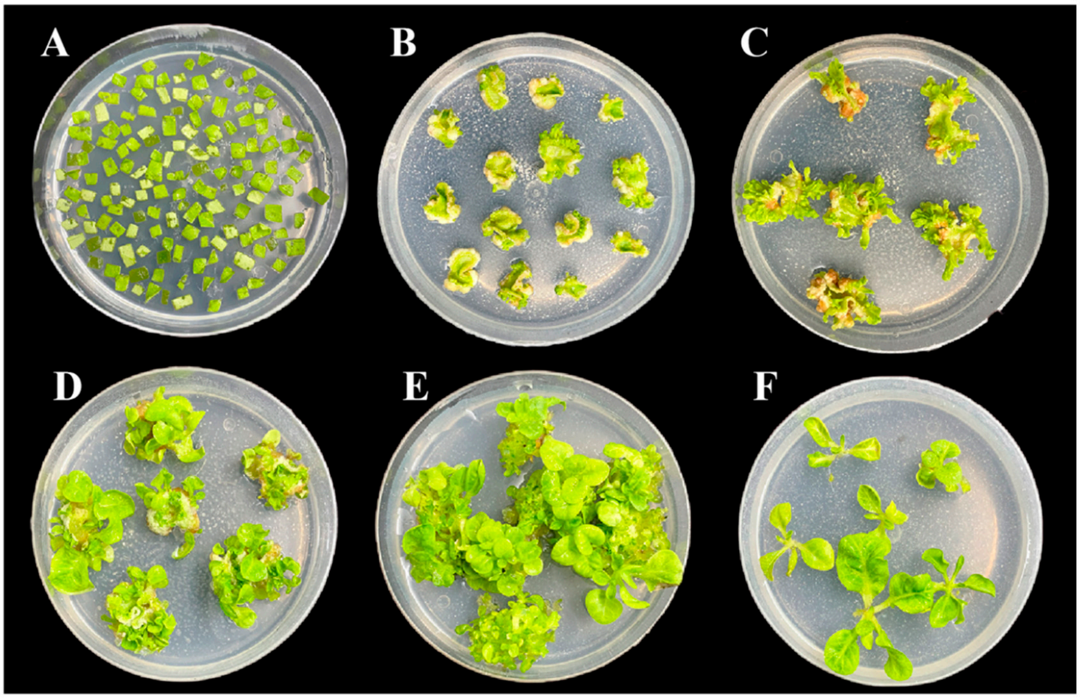

Figure S3. Growth process of transgenic tobacco of *PsHMGR* and *PsTPS1*. (**A**) Co-culture. (**B**) Induction. (**C, D**) Screening culture process. (**E**) Differentiation. (**F**) Rooting.

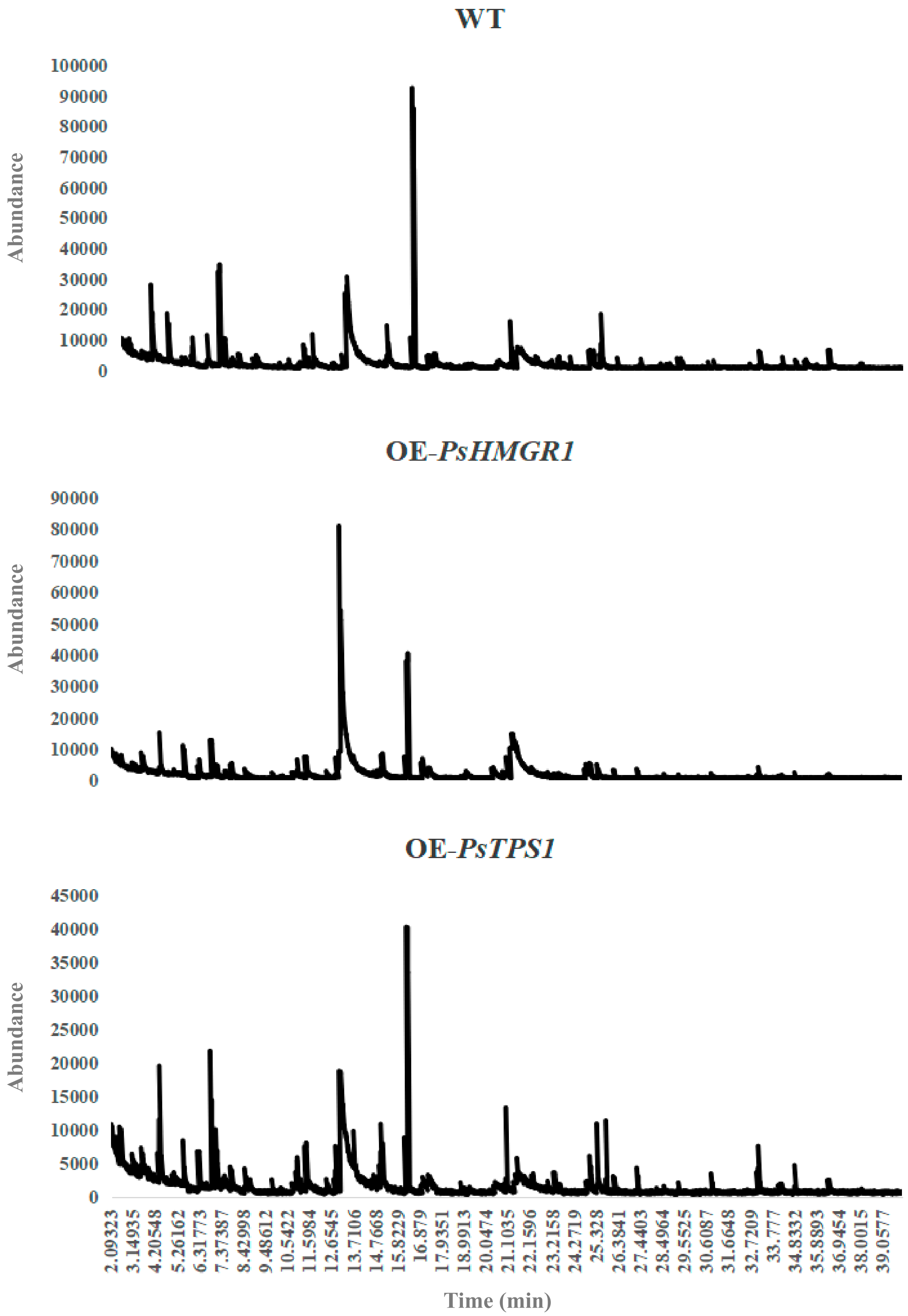

Figure S4. The GC-MS total ion chromatograms of flower from WT tobacco, transgenic lines of OE-*PsHMGR1* and OE-*PsTPS1*.

Table S1. HMGR proteins from other plant species used in phylogenetic and alignment analysis

Protein	Species	Protein ID in NCBI
DtHMGR	*Dillenia turbinata*	KAK6939125.1
TwHMGR1	*Tripterygium wilfordii*	XP_038717728.1
RcHMGR1	*Ricinus communis*	XP_002510732.1
PtHMGR1	*Populus trichocarpa*	XP_002301898.3
NnHMGR1	*Nelumbo nucifera*	XP_010266284.1
PeHMGR1	*Populus euphratica*	XP_011034792.1
MiHMGR1	*Mangifera indica*	XP_044475505.1
PaHMGR1	*Populus alba*	XP_034905451.1
PvHMGR1	*Pistacia vera*	XP_031270103.1
JcHMGR1	*Jatropha curcas*	XP_012073564.1
CaHMGR	*Chlorokybus atmophyticus*	Chrsp7S07752
MvHMGR1	*Mesostigma viride*	Mesvi286S04919
PpHMGR1	*Physcomitrella patens*	XP_024363474.1
MpHMGR	*Marchantia polymorpha*	Mapoly0015s0085.1
AsHMGR3	*Alsophila spinulosa*	Aspi01Gene13415
CrHMGR1	*Ceratopteris richardii*	KAH7280334.1
AcHMGR1	*Adiantum capillus*	KAI5058550.1
PtaHMGR1	*Pinus tabuliformis*	Pt9G14010.1
PtaHMGR9	*Pinus tabuliformis*	Pt8G45520.1
GbHMGR2	*Ginkgo biloba*	GWHGBAVD027012
AtrHMGR	*Amborella trichopoda*	XP_006849945.1
OsHMGR1	*Oryza sativa*	XM_015768351.2

ZmHMGR1	*Zea mays*	Zm00001d006040_T001
OeHMGR1	*Olea europaea*	Oeu006115.1
OeHMGR2	*Olea europaea*	Oeu035865.1
VvHMGR1	*Vitis vinifera*	VIT_203s0038g04100.1
PtHMGR2	*Populus trichocarpa*	XM_002300508.3
AtHMGR1	*Arabidopsis thaliana*	NM_106299.4
HsHMGR	*Homo sapiens*	AAG21343.1
RnHMGR	*Rattus norvegicus*	NP_037266.2
DaHMGR	*Drosophila albomicans*	XP_034115166.1
ScHMGR	*Saccharomyces cerevisiae*	ONH78258.1
GlHMGR	*Ganoderma lucidum*	ABY84849.1
LhHMGR	*Lachnellula hyalina*	XP 031002836.1
ZgHMGR	*Zobellia galactanivorans*	CAZ95248.1
SmHMGR	*Streptomyces malaysiensis*	PNG94493.1
BlHMGR	*Brevibacterium linens*	SMX84791.1

Table S2. TPS proteins from other plant species used in phylogenetic and alignment analysis

Protein	Species	Protein ID in NCBI
AtTPS02	*Arabidopsis thaliana*	NP 193406.3
AtTPS04	*Arabidopsis thaliana*	NP_564772.1
AtTPS10	*Arabidopsis thaliana*	ACF41947.1
AtTPS11	*Arabidopsis thaliana*	ABO09887.1
AtTPS14	*Arabidopsis thaliana*	NP 001185286.1
AtTPS21	*Arabidopsis thaliana*	NP_001190374.1
AtTPSGA1	*Arabidopsis thaliana*	NP_192187.1
AtTPSGA2	*Arabidopsis thaliana*	NP_178064.1
SlTPS3	*Solanum lycopersicum*	AEM05853.1
SlTPS4	*Solanum lycopersicum*	AEM05854.1
SlTPS9	*Solanum lycopersicum*	AEM05858.1
SlTPS10	*Solanum lycopersicum*	XP_004231365.1
MtTPS1	*Medicago truncatula*	AAV36464.1
MtTPS2	*Medicago truncatula*	AAV36467.1
MtTPS3	*Medicago truncatula*	AAV36466.1
AgTPSD2	*Abies grandis*	sp|O24474.1|
AgTPSD3	*Abies grandis*	O24475.1
LfTPS01	*Liquidambar formosana*	AIO10964.1
MaTPS	*Melia azedarach*	KAJ4726169.1
PaTPS (-)-germacrene D synthase	*Populus alba*	TKS02452.1
PdTPS2	*Paeonia delavayi*	UZT70947.1
PdTPS5	*Paeonia delavayi*	UZT70950.1

PlTPS	*Paeonia lactiflora*	ANA91932.1
VrTPS (-)-germacrene D synthase -like	*Vitis riparia*	XP_034678034.1
VvTPS (-)-germacrene D synthase	*Vitis vinifera*	NP_001268213.1
RhTPS32	*Rosa hybrida*	hsxptRc 038117 (Li et al., 2024)
RhTPS39	*Rosa hybrida*	hsxptRc 044988 (Li et al., 2024)
RhTPS16	*Rosa hybrida*	hsxptRc_017496 (Li et al., 2024)
RhTPS18	*Rosa hybrida*	hsxptRc_027824 (Li et al., 2024)
RhTPS20	*Rosa hybrida*	hsxptRc_027837 (Li et al., 2024)
RhTPS21	*Rosa hybrida*	hsxptRc_027838 (Li et al., 2024)

Table S3. The substance identification of GC-MS

Time	Substance	Area Percent (%)		
		WT	OE-*PsHMGR1*	OE-*PsTPS1*
2.5581	Butanal	0.7661	0.469	1.4724
3.5864	Silanediol, dimethyl-	3.8384	1.5044	2.3539
4.412	Cyclopropane, 1,1-dimethyl-	2.451	1.6661	4.5391
5.1083	3-Buten-1-ol, 3-methyl-	0.4192	0.2355	0.6012
5.5883	4-Ethylbenzoic acid, 3-methylbutyl ester	0.98	0.9301	1.3126
6.3086	Cyclotrisiloxane, hexamethyl-	1.7195	1.0022	1.4305
6.9031	1-Pentanol, 4-methyl-	4.9179	1.7122	4.7524
7.1578	Cyclopentane, methyl-	1.4845	0.5516	2.2687
7.8514	Benzene, 1,3-dimethyl-	0.8877	0.9384	1.3883
8.5321	p-Xylene	0.3803	0.5196	1.1248
9.8456	(1R)-2,6,6-Trimethylbicyclo[3.1.1]hept-2-ene	0.1609	0.239	0.3304
10.7896	s-Hydroxymethylthiobenzoate	0.3039	0.4707	0.3939
11.0081	Cyclotrisiloxane, hexamethyl-	1.05	0.7401	0.9553
11.2318	Acetic acid, octyl ester	0.5193	0.3057	0.3961
11.432	Cyclotetrasiloxane, octamethyl-	2.6511	1.1724	1.507
12.4324	Benzene, 1,4-dichloro-	0.2271	0.4099	0.6565
12.8488	1-Hexanol, 2-ethyl-	0.8344	0.9631	1.924
13.0375	Benzyl alcohol	24.0109	44.7931	26.6157
14.6646	1,3-trans,5-cis-Octatriene	0.7816	0.3896	0.6157
14.8665	Methyl valerate	0.2788	0.3078	0.7607
15.0151	4-Trimethylsilyl-9,9-dimethyl-9-silafluorene	2.94	3.1883	2.3523

15.104	Linalool	2.2028	2.8081	2.1329
16.2065	Cyclopentasiloxane, decamethyl-	10.9133	5.9928	8.6532
17.0132	Acetic acid, phenylmethyl ester	1.1806	1.7793	0.8808
17.348	Benzoic acid, ethyl ester	0.7472	0.7636	0.5353
17.5863	3-Methylenecycloheptene	--	0.1279	--
18.8483	Benzoic acid, 4-methyl-2-trimethylsilyloxy-, trimethylsilyl ester	0.126	0.0504	0.2442
20.3677	2-Propenal, 3-phenyl-	--	1.0997	--
20.5105	7-Oxo-1,3,5-cycloheptatriene-1-carbonitrile	--	0.1221	0.5428
21.0334	Cyclohexasiloxane, dodecamethyl-	2.2122	1.1538	2.8993
21.4198	2-Propen-1-ol, 3-phenyl-	5.5416	11.9317	4.1951
22.2677	Pyridine, 3-(1-methyl-2-pyrrolidinyl)-, (S)-	1.9092	1.8723	2.0199
23.023	4-Ethynyl-6,8-dioxabicyclo[3.2.1]oct-2-en-4-ol	0.3605	0.21	0.2045
23.346	3,6-Dioxa-2,4,5,7-tetrasilaoctane, 2,2,4,4,5,5,7,7-octamethyl-	0.4185	0.122	0.3445
23.5386	Heptanoic acid, phenylmethyl ester	0.2978	0.2633	0.3182
23.6662	Butane, 2,2-dimethyl-	0.2577	0.1391	0.2447
23.8972	Decanal	0.5654	0.1837	--
24.8534	Acetic acid, cinnamyl ester	2.0534	1.7249	1.0701
25.0651	6-Methyladenine, TMS derivative	0.7899	0.7443	1.328
25.3906	Cycloheptasiloxane, tetradecamethyl-	3.2027	0.5644	2.0704
25.4611	2-Propenoic acid, 3-phenyl-, ethyl ester, (E)-	--	0.6635	0.3742
25.8164	Germacrene D	--	--	7.1075

26.2035	Sulfurous acid, decyl 2-pentyl ester	0.3883	--	0.632
27.3229	Trisiloxane, octamethyl-	0.259	0.2908	0.6998
28.28	Cyanic acid, ethyl ester	0.1681	0.1198	--
28.5966	5H-Tetrazol-5-amine	0.1686	0.1683	0.1784
30.8779	4-Hydroxybenzyl alcohol, 2TBDMS derivative	0.3151	0.1975	0.6097
33.103	2-Ethylhexyl salicylate	1.1412	0.6444	1.9622
34.108	3-Chloropropane-1,2-diol, bis(tert-butyldimethylsilyl) ether	0.1135	0.0421	0.174
34.2112	1,2-Benzenedicarboxylic acid, bis(2-methylpropyl) ester	0.4778	0.1824	0.2718
34.8568	Cyclopropane, 1,1-dimethyl-2-(2-methyl-1-propenyl)-	0.3917	0.2515	1.0033
36.47	Phthalic acid, 5-methylhex-2-yl butyl ester	1.194	0.2224	0.3756
--	others	11.0013	3.0551	1.1761
